# Beyond vaccine hesitancy: Vaccine fatigue and sustained engagement with vaccination

**DOI:** 10.1016/j.pmedr.2026.103669

**Published:** 2026-10-16

**Authors:** Niharika Rustagi, Eugene Tay, Tikki Pangestu, Nick Sevdalis, Tippi Mak

**Affiliations:** Centre for Behavioural and Implementation Science Interventions (BISI), Yong Loo Lin School of Medicine, National University of Singapore, Singapore, 10 Medical Drive, MD11, Level 2, Clinical Research Centre, 117597, Singapore

**Keywords:** Vaccine fatigue, Vaccine hesitancy, Message fatigue, Information overload, Adult immunisation, Preventive behaviour

## Abstract

**Objective:** To explore vaccine fatigue as a potentially important psychological state which can affect sustained engagement and adherence with vaccination. **Methods:** This commentary integrates emerging vaccine fatigue literature with established behavioural science literature on message fatigue, information overload, and information avoidance. **Results:** We propose vaccine fatigue as a potential dynamic psychological state of exhaustion and declining motivation to continue engaging with vaccination decisions, including for individuals broadly supportive of vaccines. The model separates antecedents, including decision and information burden, from the construct of fatigue and from downstream behaviours such as avoidance, postponement, and reduction in uptake. Vaccine fatigue is positioned as complementary to, rather than synonymous with, vaccine hesitancy, and criteria are set out for distinguishing it from declining perceived benefit and from system-level changes in vaccine access. **Conclusions:** Examining vaccine fatigue as a construct, with its own research agenda and measurement approaches, could improve understanding and tailoring of interventions that sustain engagement with vaccination.

Vaccination programmes require sustained engagement. As more recommended vaccines are being introduced across the life course, greater complexity is added with different antigens, schedules, eligibility and subsidies (White et al., 2024; WHO, 2026). Individuals and caregivers face an increasing number of vaccination decisions. During the COVID-19 pandemic, population groups were asked to consider repeated boosters while simultaneously navigating oft-changing evidence and eligibility criteria. Immunisation programmes therefore face a behavioural challenge that extends beyond achieving acceptance: how to maintain engagement across complex and frequent vaccination decisions.

Existing frameworks have substantially advanced understanding of vaccine hesitancy and acceptance by focusing on confidence, perceived need, practical barriers, and other behavioural and social drivers (WHO, 2022; MacDonald, 2015). However, they tend to provide fewer insights into population groups, including those who remain broadly supportive of vaccination, who may progressively disengage from the increased frequency of vaccine recommendations. Emerging literature has categorised this as “vaccine information fatigue” or “vaccine fatigue”, but definitions and explanations remain inconsistent and can conflate antecedent burden, psychological states, and behavioural outcomes (White et al., 2024; Su et al., 2022; Stamm et al., 2023). For example, vaccine fatigue has been inferred from participants' vaccination readiness across hypothetical vaccination scenarios, rather than directly measured as a subjective psychological state (Stamm et al., 2023). Hence, a key question is not whether vaccine fatigue substitutes hesitancy, but whether it captures a separate pathway to disengagement that existing instruments do not measure.

## Vaccine fatigue as a pathway to disengagement

We propose conceptualising vaccine fatigue as a dynamic psychological state marked by exhaustion and declining motivation to continue engaging with vaccination-related decisions after repeated exposure to vaccination demands. This definition deliberately distinguishes the construct from both its antecedents and its behavioural consequences.

A growing number of vaccination recommendations can impose recurring decision, information, and administrative demands, requiring individuals to process new recommendations, engage with changing evidence, consider different sets of risks and benefits, and act on updated eligibility and immunisation schedules. Research on message fatigue and information overload suggests that repeated exposure to similarly themed health messages produces exhaustion and weariness, and that complex information environments impair effective engagement (So et al., 2017; Eppler and Mengis, 2004). Information avoidance could then become a response when further processing is experienced as effortful or aversive (Golman et al., 2017). These theories therefore make a fatigue pathway plausible, but do not establish that repeated vaccination demands would inevitably produce such a state. The proposed mechanism should therefore be approached as a research agenda to be examined, rather than assumed.

Susceptibility to this pathway is also unlikely to be uniform. The same vaccination demands may be experienced as more or less burdensome depending on individuals' prior vaccination experiences, perceived relevance and benefits of vaccination, trust in health authorities, and the cognitive or practical resources available for processing changing recommendations. Su and colleagues, for example, identified frequent immunisation demands, adverse effects, changing recommendations, low perceived disease severity, and low trust as potential contributors to vaccine fatigue (Su et al., 2022). More broadly, socioeconomic circumstances and the information environment may shape how people engage with vaccination information and recommendations (Stamm et al., 2023; Yuanita et al., 2026). These factors should therefore be investigated as potential moderators of perceived burden and fatigue, rather than assumed to constitute fatigue themselves.

As illustrated in Fig. 1, recurring or complex vaccination demands might increase perceived decision and information burden, contributing to exhaustion and declining motivation. Fatigue may then manifest as information avoidance, postponement, non-response to reminders, or disengagement, ultimately preventing or delaying vaccination. This sequence distinguishes the psychological state of fatigue from its behavioural consequences: avoidance and reduced uptake are potential manifestations of fatigue, rather than components of the construct itself. This distinction also allows vaccine fatigue to be measured independently rather than inferred from vaccination behaviour alone. A similar distinction has been operationalised in pandemic fatigue research, where Lilleholt et al.(2023) developed a scale to measure subjective exhaustion and demotivation separately from adherence behaviours (Lilleholt et al., 2023).

**Fig. 1:**
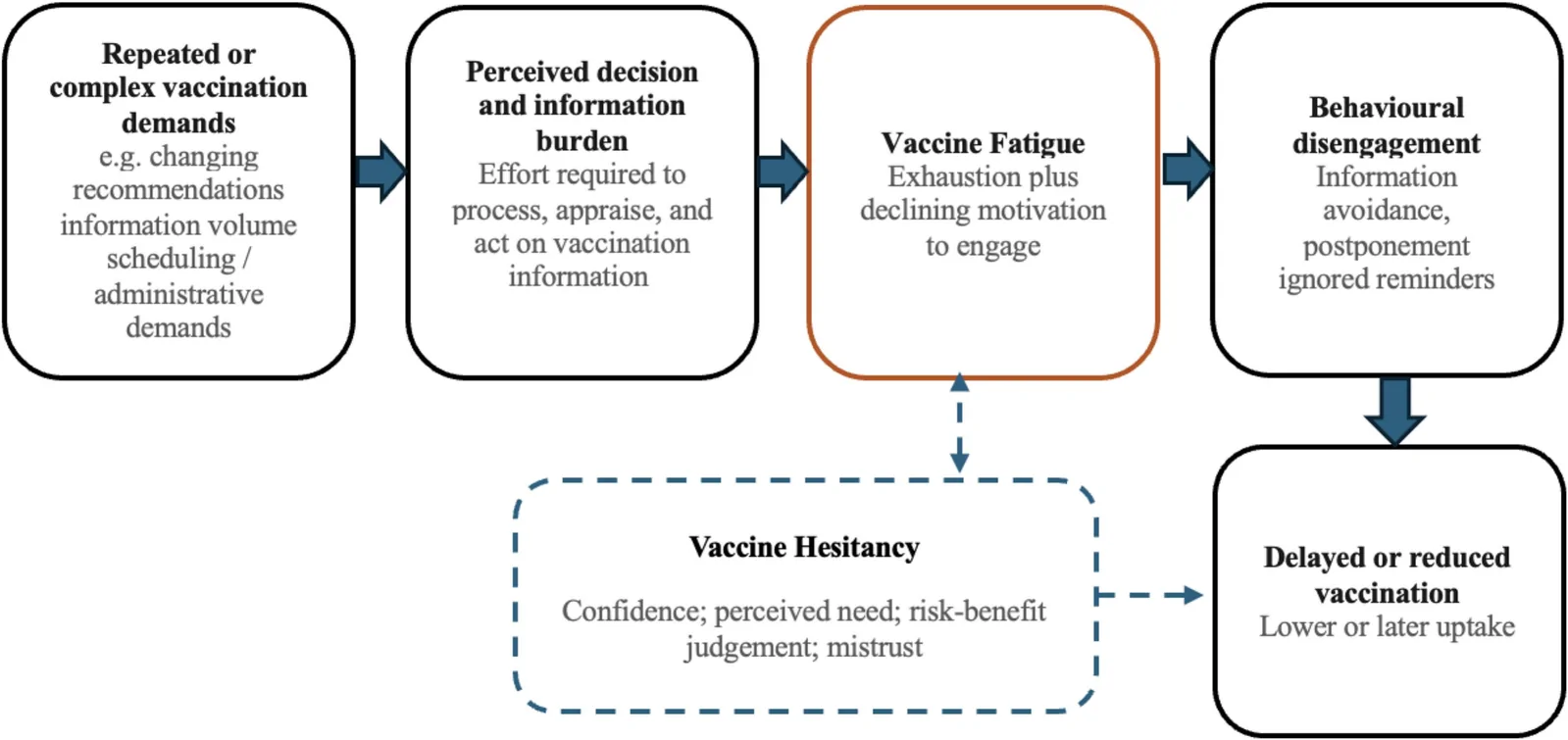
The proposed conceptual model, with antecedents, vaccine fatigue, and behavioural consequences, is denoted by solid arrows. The proposed vaccine hesitancy pathway and hypothesised reciprocal relationships with vaccine fatigue are shown by dashed arrows. These proposed pathways, their direction and temporal order require empirical testing.

## Fatigue is not simply hesitancy

Vaccine fatigue and vaccine hesitancy may have potentially interacting but distinct pathways, which would need further study. Hesitancy has been defined as reluctance or uncertainty about whether vaccination is warranted, reflecting confidence, complacency, convenience, risk-benefit judgements, or mistrust (MacDonald, 2015). Against this broader evidence base, findings from specific settings also point to the potential role of socioeconomic circumstances. For example, among people living with HIV in Indonesia, Yuanita et al. found that lower income was independently associated with hesitancy towards a hypothetical future tuberculosis vaccine (Yuanita et al., 2026). Fatigue, by contrast, concerns declining willingness to continue investing effort in vaccination decisions after repeated demands, and may occur even among individuals who remain broadly supportive of vaccination. Su et al. similarly describe fatigue as a potentially transitory state that may occur among people holding pro-vaccination views (Su et al., 2022). In simpler terms, hesitancy asks, “Should I receive this vaccine?” Fatigue asks, “Will I continue engaging with repeated vaccination decisions?”

Their relationship need not be unidirectional. Pre-existing uncertainty or mistrust could make recurring vaccination decisions more effortful and thereby increase susceptibility to fatigue. Conversely, sustained fatigue and avoidance could reduce engagement with vaccination information and potentially allow uncertainty to persist or increase. Su et al. have proposed that fatigue could progress towards more persistent forms of vaccine non-adoption, but longitudinal evidence establishing such temporal pathways remains lacking (Su et al., 2022). We therefore treat coexistence and reciprocal reinforcement as hypotheses rather than assuming that either fatigue or hesitancy necessarily causes the other.

This distinction becomes meaningful only if it generates falsifiable predictions. A valid fatigue construct should be observable among individuals who retain confidence in vaccination; change within individuals across repeated decision cycles; and predict disengagement after accounting for hesitancy, perceived benefit, convenience, and access barriers. It should also be distinguishable from these alternative explanations by the pattern of disengagement observed. Declining engagement accompanied by a reduction in perceived need or benefit would be more consistent with a change in risk-benefit judgement than with fatigue. Disengagement that resolves when practical barriers to vaccination are removed would instead be more consistent with an access constraint. Vaccine fatigue should be characterised by exhaustion and declining motivation to continue engaging even when underlying attitudes towards vaccination remain broadly favourable, perceived benefit remains relatively stable, and vaccination remains accessible. It should also respond to interventions that reduce decision burden even when vaccine beliefs themselves do not change. Longitudinal studies should additionally test whether hesitancy precedes fatigue, fatigue precedes hesitancy, or both vary independently across repeated vaccination decisions.

## Why the distinction matters for preventive medicine

Recognising vaccine fatigue is crucial because different mechanisms require different interventions. When reluctance is driven by safety concerns or mistrust, trust building and risk communication may be more appropriate. When people remain supportive of vaccines but are tired of repeated decisions, adding more persuasive information could increase rather than reduce the effort required for engagement. Interventions should instead test ways to make communication simpler and more consistent, reduce the number of steps and decisions required to receive vaccination, integrate vaccination into routine care, streamline scheduling, and use reminders or default appointments where appropriate and not excessively.

Measurement should follow the same reasoning. Existing measures of message fatigue (So et al., 2017) and the pandemic fatigue scale (Lilleholt et al., 2023) offer validated starting points, but neither captures the decision and administrative demands specific to repeated vaccination. Future scales should assess exhaustion and declining motivation to engage, while measuring antecedent burden, information avoidance, access barriers, and vaccination behaviour separately. This would allow researchers to test whether fatigue adds explanatory value beyond established constructs rather than simply re-categorising low uptake.

As vaccination becomes a recurring behaviour across the life course, maintaining engagement could be as important as achieving initial acceptance. Vaccine fatigue is most useful not as another label for non-vaccination, but as a hypothesis about why some individuals, including those who may be supportive of vaccination, progressively disengage. Distinguishing antecedents, fatigue, and behavioural consequences provides a clearer basis for more accurate measurement, mechanism-matched intervention, and preventive programmes designed for sustained vaccination uptake.

## Declarations

This manuscript is original, has not been published previously, and is not under consideration elsewhere. All authors have approved the manuscript and its submission to Preventive Medicine Reports. All authors attest they meet the ICMJE criteria for authorship. The authors declare no competing interests, and the work received no specific funding. No new data were generated or analysed; ethical approval and informed consent are therefore not applicable.

## CRediT authorship contribution statement

**Niharika Rustagi:** Writing – review & editing, Writing – original draft, Investigation, Conceptualization. **Eugene Tay:** Writing – review & editing, Writing – original draft, Investigation, Conceptualization. **Tikki Pangestu:** Writing – review & editing. **Nick Sevdalis:** Writing – review & editing, Supervision. **Tippi Mak:** Writing – review & editing, Supervision.

## Declaration of competing interest

The authors declare that they have no known competing financial interests or personal relationships that could have appeared to influence the work reported in this paper.

## Data Availability

No data was used for the research described in the article.
